# Hardware Removal in Maxillofacial Trauma: A Retrospective Study

**DOI:** 10.1155/2021/9947350

**Published:** 2021-06-23

**Authors:** Chithra Aramanadka, Abhay T. Kamath, G. Srikanth, Deepika Pai, Nishu Singla, Shriya Daundiyal, Avani Desai

**Affiliations:** ^1^Department of Oral and Maxillofacial Surgery, Manipal College of Dental Sciences, Manipal, Karnataka, India; ^2^Kasturba Medical College, Mangalore, Karnataka, India; ^3^Department of Pedodontics and Preventive Dentistry, Manipal College of Dental Sciences, Manipal, Karnataka, India; ^4^Department of Public Health Dentistry, Manipal College of Dental Sciences, Manipal, Karnataka, India; ^5^BDS Intern MCODS, Manipal College of Dental Sciences, Manipal, Karnataka, India

## Abstract

**Background:**

Miniplates are commonly used for the fixation of maxillofacial fracture segments. Removal of the hardware is controversial. A retrospective study of medical records was performed to observe the reasons for plate removal.

**Materials and Methods:**

A 10-year retrospective study of medical records was performed. Demographics, type of fracture, location, type of miniplate used, the time gap between the insertion and removal, and causes of hardware removal were assessed.

**Results:**

About 1472 patients had undergone internal fixation for the facial fractures. Stainless steel miniplate was used in 489 patients, and titanium was used in 983 patients. Out of the 42 cases, 22 cases involved the removal of titanium hardware and 20 patients involved the removal of stainless steel hardware. Infection/osteomyelitis was the main cause of hardware removal. The maximum amount of hardware failure was in the mandible. 78.6% of hardware removal was performed in males.

**Conclusion:**

Based on our study, routine removal of titanium miniplates can be performed in children to avoid growth disturbances, not indicated in adult patients unless symptomatic.

## 1. Introduction

Management of maxillofacial trauma has proved the best results by internal fixation. The available types of internal fixation methods are macroplates, miniplates, and microplates. The materials used for miniplates are either titanium or stainless steel. The main advantages of internal fixation are prompt stabilization and pain-free mastication. In recent years, titanium miniplates have gained popularity over other modalities. However, the use of plates is not without complications. They cause obstacles to the future imaging procedure when left in place. Screw loosening, palpable plate, and plate fracture will require a second surgical procedure for plate removal. Infected hardware causes localized abscess, fistula formation, nonunion, and osteomyelitis.

As reported in the literature, stainless steel or cobalt chrome plates can cause corrosion, metal allergy, toxicity, or malignant transformation [1]. Titanium and its alloy have the highest biocompatibility, excellent ductility, tensile strength, nontoxic, and resistance to corrosion than other metals [2–4]. These properties are because of the passive layer of self-regenerating oxide surface called titanium dioxide layer [5]. Titanium plates do not interfere with CT scan, MRI, or radiography. These properties make titanium popular to use.

The purpose of this study is to review the patients who have undergone plate removal postmaxillofacial trauma at our hospital.

## 2. Materials and Methods

For this retrospective study, records of patients who had undergone hardware removal over a period of 10 years (1999–2019) were collected after obtaining institutional ethical committee approval.

Medical records were screened to only include patients who required plate removal postopen reduction and internal fixation for maxillofacial trauma treated using stainless steel and titanium hardware.

Plate removal postorthognathic surgery or any other causes were not included in this study.

Data collected included age, sex, chief complaint, a history of presenting illness, medical history, history of the accident, duration of surgery from the date of fracture, type of anesthesia used (LA/GA), tooth in line of fracture, and tooth extracted if any.

The fractures were divided as a type of fracture, location of fracture, and number of fractures.

The cause of hardware removal was then classified as removal due to screw loosening/hardware failure (plate or screw fracture)/local osteomyelitis/plate exposure/any other.

The correlations between the causes for hardware removal and the associating factors were then determined using appropriate statistical analysis.

## 3. Results

From the year 1999–2019, 42 patients with a history of craniomaxillofacial fractures secondary to trauma underwent hardware removal. Out of the 42 patients, 33 (78.6%) were male and nine (21.4%) were female. The maximum amount of plate removal was seen in the patients of the age group 31–40 years (35.7%) and the least was for patients < 10 years of age (4.8%).

The most common cause of trauma was RTA (road traffic accident) which was seen in 37 (88%) of the patients and the rest of the five (12%) patients were injured due to a fall (Table 1).

The location of fracture for 19 (45.2%) patients was the mandibular region and for six (14.3%) patients was the maxillary region. For four (9.5%) patients, the fracture was in the zygomatic region (Table 1).

Out of the 42 patients, the pus discharge was the chief complaint given by 17 (40.4%) patients. Four (9.5%) underwent elective hardware removal which was prophylactic in a growing patient. Two (4.8%) patients complained of heaviness and metallic feeling in the mouth. One (2.4%) patient complained of loss of sensation. One (2.4%) patient complained about the mobility of the fracture segment (Table 2).

There was a higher incidence of hardware removal within seven months to one year which involved 12 (28.5%) patients (Table 3).

Out of the 42 cases, 22 cases involved the removal of titanium hardware (52.4%) and 20 patients involved the removal of stainless steel hardware (47.6%) (Table 4).

In nine cases, the tooth was in the line of the fracture and was extracted (21.4%).

GA (general anesthesia) was used in 25 cases (59.5%), and LA (local anesthesia) was used in 17 cases (40.5%). The intraoral approach was used in 34 cases (81%), extraoral approach in seven cases (16.6%), and the combined approach in one case (2.4%).

The association between the indication of hardware removal specifically soft tissue infection/osteomyelitis and the type of hardware used was found to be statistically significant (*p* < 0.05).

The correlation between the indication of hardware removals such as exposed hardware and screw loosening and the type of hardware used was not statistically significant.

This correlation was determined using the chi-square test.

Certain observations made in our study were as follows:

1. Soft tissue infection/osteomyelitis was the main cause of hardware removal in patients with stainless steel hardware (55%)
2. Only one case required hardware removal due to nerve injury
3. The maximum amount of hardware failure was in the mandible
4. 78.6% (33 cases) hardware removal was performed in males

## 4. Discussion

Plate removal in patients is controversial; in some cases, surgical removal is performed only if there are clinical symptoms, and in some cases, it is performed as a routine procedure after the patient has been explained about the risks and informed consent is taken [6–12]. Some authors believe that the plate can act as a foreign object. This might cause a restriction in growing patients which is a factor to be considered [12, 13]. It is important to note that removal of the plate does not imply failure of treatment [6]. Symptoms may develop after the bony union has occurred in when the plate acts as a foreign body and leads to complications that may necessitate the removal of the plate [14].

This 10-year retrospective study was performed to determine the reasons for hardware removal in patients who had undergone open reduction and plate fixation postcraniomaxillofacial fractures secondary to trauma. A total of 1,472 patients had undergone internal fixation for facial fractures. Stainless steel miniplate was used in 489(33%) patients, and titanium was used in 983(66%) patients. Of the 42 patients who required plate removal, the most common cause of trauma in these patients was road traffic accident and 33 (78.6%) were males. This could be attributed to the fact that young males are more prone to be involved in RTA's [15].

During the study, we noticed that from the year 1999 to 2011, stainless steel plates were most commonly used, and from 2011 to 2019, titanium miniplates were popularly used. This may be due to the unavailability of titanium in the early period. Though stainless steel plates are less expensive, the disadvantages include less durability, bulky, cause radiographic scatter at MRI, galvanic potential, and more reactive than titanium and show increased rate of infection [14, 16]. Titanium plates have improved biocompatibility when compared to stainless steel plates and excellent osseointegration along with high corrosion resistance [14, 16]. A study showed that corrosion in titanium plates is caused mainly due to manufacturing or handling of the materials by the surgeon, and if these two factors are kept in mind during the placement, then long-term stability of titanium is guaranteed, and routine removal is not required in asymptomatic patients [12]. We do agree the handling errors do occur while drilling the holes and contouring the plate, especially with the unexperienced hands in educational institutions.

In our study, a higher number of plates were removed from the mandibular region. Out of 42 patients, 24 patients underwent plate removal from the mandibular region. Mandible fractures are due to severe traumatic forces which lead to further complications which can explain the increased number of hardware removal in this region [17]. Contributing factors for hardware failure in the mandibular region can be the mobility of the mandible which interferes with the stability of the prosthesis, inability to maintain proper oral hygiene, collection of saliva or food in the vestibule [17], proximity to tooth roots [18], constant irritation to the surrounding tissues [18], and trauma due to continuous masticatory forces [10]. These can cause infections and eventually failure and removal of hardware in this region. Nerve injury might also lead to hardware removal in some cases if the plates were placed in close proximity to the nerves in the mandible [18–20]. Other factors can also be the inability to maintain routine follow-up which might lead to improper follow-up measures and local infection [18, 21, 22].

Our study showed that infection was the most commonly associated complication leading to hardware failure in trauma patients. This was similar to conclusions made in other studies [8–12, 14, 15, 17, 18, 20–23]. The causes may be lack of blood supply due to comminuted fractures with periosteal detachment [10], inability to maintain proper oral hygiene [15, 18, 21, 23], increased exposure of the fractured site to the oral environment [1, 24], time duration between the accident and the surgery [15, 21], smoking [12, 23], repeated trauma due to mastication and dentures [10], delayed healing [22], infected tooth [14], improper fracture diagnosis [18], lack of stability causing movement of the plate which acts as a foreign body [15, 18, 19], and intraoral approach causing lack of proper visibility [15, 18, 23].

Another cause of hardware failure in our study was hardware exposure and palpable hardware which was commonly seen in the zygomatic and infraorbital region. This could be attributed to the zygomaticofrontal region, and the infraorbital region has thin skin, and this leads to the screws becoming more visible over time [17]. Exposure might be commonly seen in cases with thin, soft tissue covering over the plates. Continued exposure leads to palpable hardware and secondary infection [7].

Hardware loosening is also an indication for plate removal during the healing period which can be treated by replacement of the hardware or by rigid external fixation [7].

Increased incidence of plate removal was seen in patients who underwent open reduction and internal fixation with an intraoral surgical approach [15, 18, 23]. Our study showed that this was five times more than those who underwent open reduction and internal fixation using the extraoral approach. This may be because of the fact that the thickness of soft tissue is much lesser to cover the foreign body. The intraoral approach leads to reduced exposure of the field along with reduced visibility which can lead to further complications such as damage to nerves, tooth roots, and improper placement of the plates which will eventually lead to an increased chance of infection, hardware failure, and removal [15, 18]. Thus, the extraoral approach can be used to ensure a more sterile environment along with increased visibility and fewer complications [15, 18, 23]. However, there is always a risk of scar formation.

Proper oral hygiene, constant follow-up, and radiographic examination of the teeth in the line of fracture followed by endodontic treatment if necessary should be performed in all cases to reduce the chance of complications [15, 18]. Stability by using adequate rigid fixation of the fracture leads to decreased mobility of the foreign body and contributes to a lower risk of infection. This is very important for mandibular fractures as the mandible is load-bearing and mobile [18].

According to the statistical analysis, our study showed that majority of the plate-related problems usually developed within the first seven months to one year. This is in line with the data observed from other studies [8, 11, 13, 14]. Thus, informed consent of the patient should be taken at the time of the initial surgery, and plate removal can be mentioned as a possible postoperative complication that may require the patient to undergo a second surgery [8, 11, 21].

A comparative study by Deepak et al. showed that more complications were seen in patients having stainless steel prosthesis when compared to titanium. This was because the time taken to adapt stainless steel plates was far greater, and this is attributed to the pliability of the material. This could be the factor leading to higher infection rates and wound dehiscence [16].

Our study also showed that the number of removal of titanium plates, when compared to stainless steel, was less. Of the 983 patients, titanium plates amounted to 22 (0.022%) cases only, and of the 489 patients, stainless steel plate removal was performed in 20 (0.04%) cases. This is similar to the data seen in other studies [8, 16]. Studies have also shown that titanium plates do not require routine removal unless clinically indicated [6, 9–12]

## 5. Conclusion

Based on the results seen in our study, most of the cases showed complications within the first year of placement irrespective of the material used. Thus, routine removal is to be considered when the bony union has occurred to prevent any disturbance in the growth of the facial bones in children. Hence, introducing the protocol for routine removal can be a preventive measure, which requires a second surgical procedure. Our current protocol is the removal of titanium plates only in symptomatic adult patients as titanium is most biocompatible and has osseointegration properties.

## Figures and Tables

**Table 1:** History and location of fractures.

Variable	Categories	*n* ^*∗*^	%
History of trauma	Road traffic accident (RTA)	37	88
	Fall	5	12

Location of fracture	Mandibular	19	45.2
	Maxillary	6	14.3
	Maxillary and mandibular	2	4.8
	Zygomatic	4	9.5
	Maxillary and zygomatic	2	4.8
	Mandibular and zygomatic	1	2.4
	Panfacial	7	16.7
	Lefort 3	1	2.4
	**Total**	42	100

**Table 2:** Chief complaint of study subjects.

Variable	Categories	*n* ^*∗*^	%
Chief complaint	Pain with inability to open mouth/chewing food/discomfort	10	23.9
	Pain with pus discharge/swelling/sinus opening	17	40.4
	Exposed/palpable hardware	7	16.6
	Elective/prophylactic removal in growing patient	4	9.5
	Feeling of heaviness/feeling of mental in mouth	2	4.8
	Loss of sensation	1	2.4
	Mobility of fracture segment	1	2.4

**Table 3:** Time of hardware removal of study subjects.

Variable	Categories	*n* ^*∗*^	%
Time of hardware removal	1 day–1 week	2	4.8
	1 month–6 months	9	21.4
	7 months–1 year	12	28.5
	>1 year-2 years	5	11.9
	>2 years-3 years	5	11.9
	4 years-5 years	5	11.9
	7 years	2	4.8
	11 years-12 years	2	4.8

	Total	42	100

**Table 4:** Type and process of removal of hardware of the study subjects.

Variable	Categories	*n* ^*∗*^	%
Type of hardware removed	Stainless steel (SS)	20	47.6
	Titanium (ti)	22	52.4

Type of plate based on the plate thickness	1 mm	12	28.6
	1.5 mm	20	47.6
	2 mm	8	19
	2.5 mm	2	4.8

Anaesthesia	General anesthesia (GA)	25	59.5
	Local anesthesia (LA)	17	40.5

Approach	Intraoral	34	81
	Extraoral	7	16.6
	Combined	1	2.4

Tooth involved	Yes	9	21.4
	No	33	78.6

Tooth extracted	Yes	9	21.4
	No	33	78.6
	**Total**	42	100

^*∗*^ *n* is the number of subjects.

## Data Availability

The data used to support the findings of this study are available from the corresponding author upon request.

## References

[1] Kubota Y., Kuroki T., Akita S (2012). Association between plate location and plate removal following facial fracture repair. *Recon and Aest Surg*.

[2] Woodman J. L., Jacobs J. J., Galante J. O., Urban R. M. (1984). Metal ion release from titanium-based prosthetic segmental replacements of long bones in baboons: a long-term study. *Journal of Orthopaedic Research: Official Publication of the Orthopaedic Research Society*.

[3] Manor Y., Chaushu G., Taicher S. (1999). Risk factors contributing to symptomatic plate removal in orthognathic surgery patients. *Journal of Oral and Maxillofacial Surgery*.

[4] Jain R., Podworny N., Anderson G. I. (1997). Effects of stainless steel and titanium low contact dynamic compression plate application on the vascularity and mechanical properties of cortical bone after fracture. *Journal of Orthopaedic Trauma*.

[5] Allurkar S., Patil S. K., Joshi U. M., Shah K., Thakur N., Mangalgi A. (2016). Surface analysis of titanium maxillofacial plates and screws retrieved from patients. *Journal of Dental Research*.

[6] Mosbah M. R., Oloyede D., Koppel D. A., Moos K. F., Stenhouse D. (2003). Miniplate removal in trauma and orthognathic surgery-a retrospective study. *International Journal of Oral and Maxillofacial Surgery*.

[7] Cahill T. J., Gandhi R., Allori A. C. (2015). Hardware removal in craniomaxillofacial trauma. *Annals of Plastic Surgery*.

[8] Bhatt V., Langford R. J. (2003). Removal of miniplates in maxillofacial surgery: university Hospital Birmingham experience. *Journal of Oral and Maxillofacial Surgery*.

[9] Matthew I. R., Frame J. W. (1999). Policy of consultant oral and maxillofacial surgeons towards removal of miniplate components after jaw fracture fixation: pilot study. *The British Journal of Oral & Maxillofacial Surgery*.

[10] Rallis G., Mourouzis C., Papakosta V., Papanastasiou G., Zachariades N. (2006). Reasons for miniplate removal following maxillofacial trauma: a 4-year study. *Journal of Cranio-Maxillofacial Surgery*.

[11] Bhatt V., Chhabra P., Dover M. S. (2005). Removal of miniplates in maxillofacial surgery: a follow-up study. *Journal of Oral and Maxillofacial Surgery*.

[12] O’Connell J., Murphy C., Ikeagwuani O., Adley C., Kearns G. (2009). The fate of titanium miniplates and screws used in maxillofacial surgery: a 10 year retrospective study. *International Journal of Oral and Maxillofacial Surgery*.

[13] Bakathir A. A., Margasahayam M. V., Al-Ismaily M. I. (2008). Removal of bone plates in patients with maxillofacial trauma: a retrospective study. *Oral Surgery, Oral Medicine, Oral Pathology, Oral Radiology, and Endodontology*.

[14] Hernandez Rosa J., Villanueva N. L., Sanati-Mehrizy P., Factor S. H., Taub P. J. (2016). Review of maxillofacial hardware complications and indications for salvage. *Craniomaxillofacial Trauma & Reconstruction*.

[15] Chaushu G., Manor Y., Shoshani Y., Taicher S. (2000). Risk factors contributing to symptomatic plate removal in maxillofacial trauma patients. *Plastic & Reconstructive Surgery*.

[16] Deepak S., Manjula S. (2011). Comparison of Titanium bone plates and screws vs. stainless steel bone plates and screws in the management of mandibular fractures – a long term clinical study. *Int. Journal of Clinical Dental Science.*.

[17] Islamoglu K., Coskunfirat O. K., Tetik G., Ozgentas H. E. (2002). Complications and removal rates of miniplates and screws used for maxillofacial fractures. *Annals of Plastic Surgery*.

[18] Murthy A. S., Lehman J. A. (2005). Symptomatic plate removal in maxillofacial trauma. *Annals of Plastic Surgery*.

[19] Nagase D. Y., Courtemanche D. J., Peters D. A. (2005). Plate removal in traumatic facial fractures. *Annals of Plastic Surgery*.

[20] Raja D. R., Madhulaxmi M., Abdul Wahab P., Jesudasan J. S. (2013). Is plate removal after orthognathic surgery mandatory?. *International Journal of Dental Sciences and Research*.

[21] Kent S. J. W., Al-Izzi T., Herbert C., Ryan M. (2017). A retrospective review of metal plate removal in an oral and maxillofacial surgery department. *International Journal of Dental Sciences and Research*.

[22] Hanson J., Lovald S., Cowgill I., Erdman M., Diamond B. (2011). National hardware removal rate associated with internal fixation of facial fractures. *Journal of Oral and Maxillofacial Surgery*.

[23] Thorén H., Snäll J., Kormi E., Lindqvist C., Suominen-Taipale L., Törnwall J. (2010). Symptomatic plate removal after treatment of facial fractures. *Journal of Cranio-Maxillofacial Surgery*.

[24] Eppley B. L., Sparks C., Herman E., Edwards M., McCarty M., Sadove A. M. (1993). Effects of skeletal fixation on craniofacial imaging. *Journal of Craniofacial Surgery*.

